# Geospatial analysis of HIV-Related social stigma: A study of tested females across mandals of Andhra Pradesh in India

**DOI:** 10.1186/1476-072X-9-18

**Published:** 2010-04-12

**Authors:** Rashmi Kandwal, Ellen-Wien Augustijn, Alfred Stein, Gianluca Miscione, Pradeep Kumar Garg, Rahul Dev Garg

**Affiliations:** 1 Indian Institute of Technology IIT, Roorkee, Roorkee-247667, India; 2 ITC International Institute for GeoInformation Science and Earth Observation, Hengelosestraat 99, P.O. Box 6, 7500 AA Enschede, the Netherlands

## Abstract

**Background:**

In Geographical Information Systems issues of scale are of an increasing interest in storing health data and using these in policy support. National and international policies on treating HIV (Human Immunodeficiency Virus) positive women in India are based on case counts at Voluntary Counseling and Testing Centers (VCTCs). In this study, carried out in the Indian state of Andhra Pradesh, these centers are located in subdistricts called mandals, serving for both registration and health facility policies. This study hypothesizes that people may move to a mandal different than their place of residence for being tested for reasons of stigma. Counts of a single mandal therefore may include cases from inside and outside a mandal. HIV counts were analyzed on the presence of outside cases and the most likely explanations for movement. Counts of women being tested on a practitioners' referral (*REF*s) and those directly walking-in at testing centers (*DW*s) were compared and with counts of pregnant women.

**Results:**

At the mandal level incidence among *REF*s is on the average higher than among *DW*s. For both groups incidence is higher in the South-Eastern coastal zones, being an area with a dense highway network and active port business. A pattern on the incidence maps was statistically confirmed by a cluster analysis. A spatial regression analysis to explain the differences in incidence among pregnant women and *REF*s shows a negative relation with the number of facilities and a positive relation with the number of roads in a mandal. Differences in incidence among pregnant women and *DW*s are explained by the same variables, and by a negative relation with the number of neighboring mandals. Based on the assumption that pregnant women are tested in their home mandal, this provides a clear indication that women move for testing as well as clues for explanations why.

**Conclusions:**

The spatial analysis shows that women in India move towards a different mandal for getting tested on HIV. Given the scale of study and different types of movements involved, it is difficult to say where they move to and what the precise effect is on HIV registration. Better recording the addresses of tested women may help to relate HIV incidence to population present within a mandal. This in turn may lead to a better incidence count and therefore add to more reliable policy making, e.g. for locating or expanding health facilities.

## Background

HIV related stigmas are a driving force influencing the behavior and location specific testing results of persons seeking HIV testing [1]. Much has been reported about stigmatized behavior, but little has been investigated on the possible movements of persons in general and women in particular seeking anonymity and thus moving from their residence to other places for getting tested. Misinformation about HIV testing attitudes, and HIV stigmatizing beliefs represent potential barriers to testing [2-5]. Kaplan et al [3] note that our understanding of the mechanisms by which HIV related stigma perpetuates is limited. To plan improved interventions it is necessary to better understand the behavioral pattern of those getting tested. Various population based studies report major differences from sentinel surveillance based estimates [6-8].

Hence, obtaining a good insight into the spread of the HIV incidence requires a reliable registration of those infected. Registration has an effect on the official statistics as well, as for example the Indian government recently reported a change in the official incidence value from 4.5 to 1.5% at the national level, similarly to what happened in Kenya (Appendix I). According to Pandey et al [9], the earlier HIV estimates in India were based on HIV sentinel surveillance (HSS) data. It is assumed that prevalence among attendees of antenatal clinics serves as as a proxy for the prevalence in the general population and prevalence among the patients of sexually transmitted diseases as a proxy for the prevalence among populations with high risk behavior. The absence of HIV surveillance among female sex workers and men having sex with men was a weakness of this system. Those two groups were later included in the estimation but sexually transmitted disease clinics were not discarded. This resulted in double counting. In 2006, improved data became available as the sentinel surveillance among ANC women was expanded covering nearly every district in the country allowing better geographical representation with adequate data for each state. Additionally, community based HIV prevalence measured by the National Family Health Survey-3 [10] provided an opportunity to replace earlier assumptions, validate the HSS data and improve the HIV estimate. Calculations and estimates in Pandey et al [9] reverse the number of total HIV prevalence in India. They quote that the current estimate is a revision based on improved data and methodological changes. The difference between the current estimate and previously published estimates does not represent a true decline at the population level.

Oppong [11] while discussing the data problems in HIV research quotes that sample size, nonrepresentative samples, and geographic and testing bias, tend to make seroprevalence estimates defective if generalized beyond its sample population. A change of scale to the facility level improved representativeness and lead to more promising results. Until recently, methodology was developed and applied to data that were only available at the state and the district level. The analysis presented in our study goes one step further and considers the sub-districts or mandals level.

Disease data as analyzed in this study have a clear spatial component. Registration is done at hospitals and clinics that are located in mandals, the spread of the disease is most likely done by roads and transport networks and spatial components are possibly helpful to provide better estimates. To do so, geographical information system was used in this study, providing opportunities to analyze HIV data and related layers of information in a quantitative way by means of readily available spatial statistical tools. The aim of the study is to quantify the degree to which women move to a different place for HIV-testing and to find explanatory variables. To do so, data sets of women being tested on a practitioners' referral and those directly walking-in at testing centers were compared with data sets of pregnant women. HIV data used was collected in 2006 from the Indian state of Andhra Pradesh, where a well-established registration system exists.

## Methods

### Study area

**Figure 1:**
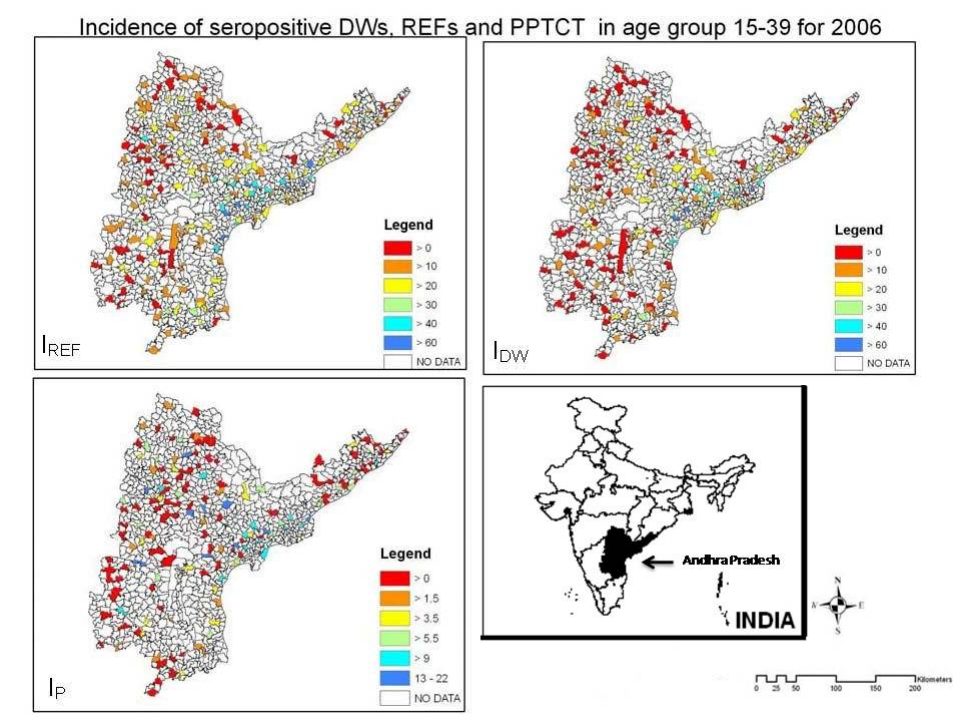
**incidence**. labelincidence Maps showing *I*_*REF*_, *I*_*DW *_and *I*_*P *_calculated per 10,000 populations in age groups 15-39. INSAT : Map of India, showing the state of Andhra Pradesh.

This study area concerns the state of Andhra Pradesh (Insat, Figure 1). For HIV, Andhra Pradesh is the second worst affected state of India, after the state of Manipur. It is in size the fifth largest state of India with an area of 277,000 km^2^, accounting for 8.4% of India's territory. It is divided into 23 districts and 1103 (2001) sub-districts called mandals. Based on the Census 2001, the total population is 76 million, making it the fifth most populous state, with a population density of 277 people km^2^. Population is mainly rural (approximately 72.7%). Andhra Pradesh is predominantly an agriculture-oriented economy, e.g. it is the largest producer of rice in India. The movement of agricultural products and raw and finished material depends on road transport [12]. Because of its large population, good data accessibility, well established e-governance and its comparatively better health infrastructure it is well suited for this study.

### Data Used

Population data from the national census in 2001 have been used. Estimation of the annual projected population for 2006 has been based on the population projection for India [13]. Base data to delineate the different mandals and their boundaries were made available from the National Remote Sensing Center. The Eicher Andhra Pradesh Road map ^©^2008 was used to generate the roads layer for application in the GIS. This study uses HIV data collected by the NACO (National AIDS Control Organisation) in India on indicators supplied by Voluntary Counseling and Testing Centers (VCTC)s. In 2006, there were 190 VCTCs located within 1103 mandals. These data are the most comprehensive one on the population and thus may provide clues to understand the HIV epidemiology [14]. The role of these centers as a convenient and cost effective tool for monitoring the HIV epidemic is well known. Their high coverage within the state and country is a key in the overall success in combating HIV [4,15,16]. As our study is mandal based, the data do not include mobile VCTCs and primary health care centers since these units are district based and hence can not be assigned to a single mandal. The VCTC data represent unbiased samples from the general population. They distinguish two types of clients: self-referred clients or Direct Walk-ins, (*DW*) and provider-referred clients or Refferals (*REF*). *DW*s voluntarily present themselves at a VCTC, whereas *REF*s are referred for HIV counseling by health-care providers. The decision to undergo an HIV test is voluntary for *REF*s [17].

Gynecological units are present in different hospitals and clinics to serve as a Prevention Of Parent to Child Transmission (PPTCT) center. Such units facilitate assistance to pregnant women and take measures to control the transmission of infection to the newborns [18]. 167 units were functional in Andhra Pradesh in 2006, covering approximately 10% of the pregnancies in the country. In 2006 PPTCT data were available from January to August that were used in this study.

### Incidence Calculation

This analysis is based on the representativeness of PPTCT data. Therefore, the subset of HIV-positive women from the VCTC data was selected. To make group-wise comparisons, incidence is calculated per 10,000 female population. Table 1, extracted from [10], shows that pregnant women mainly belong to the age class 15-39. HIV-positive women belonging to the same age-group were selected from the *DW *and *REF *groups in the VCTC data. Data from these three groups represent the numbers of HIV-positives belonging to age range 15-39 in a particular mandal at a given time. The projected female population for 2006 in the 15-39 age group was obtained as the percentage of females, based on the age divisions at the district level group for 2006. The total district level population (*T*_*D*_) is available in 5 year age groups (*A*_*D*_). First the population was projected for 2006, from this the percentage (*X*_*D *_= (*A*_*D *_× 100)/*T*_*D*_) per district for age range 15-39 is calculated. The female population in each mandal for this age group (*A*_*M*_) is calculated using *X*_*D *_and the total mandal population *T*_*M *_projected for 2006 as *A*_*M *_= (*X*_*D *_× *T*_*M*_)/100.

**Table 1:** fertil

Age specific fertility rates for women in Andhra Pradesh			
**Age**	**Urban**	**Rural**	**Total**

15-19	0.071	0.114	0.098

20-24	0.174	0.166	0.168

25-29	0.066	0.054	0.058

30-34	0.029	0.017	0.021

35-39	0.006	0.010	0.009

40-44	0.001	0.004	0.003

45-49	0.000	0.000	0.000

Incidences per 10,000 inhabitants, denoted as *I*_*P*_, *I*_*DW *_and *I*_*REF *_for pregnant women, *DW*s and *REF*s, respectively, with subscripts denoting the related group, are determined as(1)

### spatial analysis

Methods for this study are based on a spatial pattern analysis, outlier detection and establishment of spatial relationships. An outlier is defined as an unusually high or low HIV frequency as compared to the *DW *and *REF *data sets for the same mandal and/or values for the same data set for other mandals in the direct vicinity. The first question considered is which women are represented by the three data sets and what is their behavior and spatial distribution? The second question is what types of movement should be linked to HIV testing and which group is expected to have a particular type of movement behavior? The spatial pattern analysis was done based on the understanding of the type of the three diverse groups and what they represent.

• Pregnant women may be used as a proxy for prevalence in the overall population [19]. They are mostly married and they are equally distributed over the population. There are well-known limitations, however, as not all pregnant women may access the antenatal care services or may accept HIV testing. This apparently is of a limited importance, because in this data set 92% of women attending the antenatal clinics accepted the tests.

• Women registered as *REF*s show a larger diversity than women registered as *DW*s. Hence more cases are expected in places with more and better facilities for testing, i.e. with the order of the facilities. *REF*s, being already asked by a practitioner to get tested, are less likely to move. So they may be more inclined go to the nearest testing center.

• Women registered as *DW*s are more likely to belong to a high risk category, i.e. being involved in sex trade and injecting drug use. Their spatial pattern may reflect the locations of areas conducive for risk activities like regions rich in trade that are well connected with urban setups, such as roads signifying movement. Because *DW*s get tested on their own, they may move to any facility of choice. Thus *DW*s have an opportunity of travelling larger distances and thus have a higher probability of being registered at another place. They might therefore seek testing at anonymous sites and hence they will form the group governing the movement.

Any difference in the spatial pattern for the three groups can be attributed to the cause of movement. Based on the scale of analysis, socio-psychologic behaviour of women getting tested and the societal setup, we distinguish accessibility movement and hierarchical movement. Accessibility movement relates facilities that are better connected to urban setups with a higher incidence. Since the choice of connection is important for the *DW*s, this type of movement should be identifiable by the highest correlation of connectivity with higher incidences of *DW*s. Hierarchical movement relates the order of the facilities, e.g. from a community health center to medical college, to a higher incidence. In particular for *REF*s higher order facilities should show a higher incidence, as they can be referred to the best facility, usually equated to the highest order. Three other types of movement that are not detectable are distinguished: random movement, movements at a very short distance (women aware of HIV usually select a VCTC within 60 km away or private clinics suggested by friends (*Pers. Comm*.)) and movements that neutralize or counterbalance movements between mandals.

Based on the above the following assumptions were made for the three groups under study:

1. Women to be tested at a VCTC in a particular mandal as *REF*s (*F*_*REF*_) comprise both the women (*F*_*RL*_) from the same mandal and women from other mandals (*F*_*RO*_) that aim to maintain their anonymity.(2)

2. Women tested as *DW*s (*F*_*DW*_) at a VCTC in a particular mandal comprise both the local walking in women (*F*_*DL*_) and women walking in from other mandals (*F*_*DO*_).(3)

3. Pregnant women (*F*_*P*_) getting tested at a PPTCT center in a particular mandal are those belonging to this mandal only. Their main incentive is to receive antenatal care and the HIV test is additional to that.

4. The proportion of local *DW*s is assumed to be less than the proportion of local *REF*s in each mandal, since the local *DW*s have an opportunity to move to other places:(4)

It is thus assumed that the local *DW*s (usually represented by the sex workers) would generally move and hence would be less represented as compared to the *REF*s who will remain at their place of stay.

Movement was analyzed first on the basis of an outlier detection scheme, showing mandals which deviate from the normal behavior. Secondly, a spatial cluster analysis was applied to detect geographic variation patterns and identifying locations having statistically significant higher incidences as compared to their neighbors [14]. Finally, spatial regression was carried out to quantify the observed patterns.

### Outlier detection

Mandals at both ends are outliers: lower-end outliers represent mandals with *I*_*P *_comparatively higher or nearly equal to *I*_*DW *_and *I*_*REF*_, whereas higher-end outliers represent mandals where *I*_*P *_is lower than *I*_*DW *_and *I*_*REF*_. Incidence maps were generated within the ArcGIS [20] environment using the *I*_*P*_, *I*_*DW *_and *I*_*REF *_incidences. These maps were used in turn to first yield two difference maps relating the *REF *and *DW *groups to the *P *group(5)

These maps were classified into six classes. Lower ranges in the *ID*_1 _and *ID*_2 _correspond to higher *I*_*P *_values. Therefore, smaller class intervals were chosen in the lower range keeping negative values as one class, and then having class ranges of 0, 2, 5 and 10, respectively. These maps could thus identify mandals with strong differences between pregnant women and women from the general population. Values equal to 0 in the *ID*_3 _map identify mandals with equal *I*_*DW *_and *I*_*REF*_. Mandals with a high *ID*_3 _value are outliers, representing an exceptionally high *I*_*REF*_.

### Spatial Cluster analysis

Spatial cluster analysis is commonly used in disease surveillance and spatial epidemiology [21]. For this study, SaTscan™ [22] software was used to compare spatial clustering in the data with a Poisson model showing randomness. In total, 1103 - 190 = 913 missing values represent mandals without facilities. To account for these, the missing value adjustment parameter was used, assigning a relative risk of zero to mandals without data. In doing so, the analysis ignores those mandals. The results of the spatial clustering in SaTscan™ were imported into the ArcGIS environment where significant clusters were visualized using *p*-values.

### Establishing spatial relationships

On the basis of the above analysis, it can be predicted if people are moving and a trend can be estimated. A hypothesis is established for the following relations to be possibly significant:

1. The effect of facility hierarchy plays a role in a higher *I*_*REF *_value in a mandal. Thus a higher order facilities will draw more *REF*s and more pregnant women than a lower order facility.

2. Vicinity of roads may increase the *I*_*DW *_because of better connectivity. Therefore, the distance to a major road may have a positive relation to *I*_*DW*_. Also the number of road intersections within a mandal represents a better connectivity that may increase the movement of *DW*s.

3. Incidence in pregnant women most likely remains unaffected by connectivity, given their status of pregnancy, whereas it may be related with the number of neighboring mandals. If a mandal has more neighboring mandals then it may be attractive to visit, realizing that many mandals do not have their own testing facility.

Therefore, effects of the following exploratory variables are investigated:

1. Type of facilities (*T*_*F*_) based on their size and strength within a mandal. These types include Community Health Centers (CHCs), with 30-50 beds and one clinical specialty, Area Hospitals (AHs), with approximately 100 beds and four clinical specialties like obstetrics & gynecology, pediatrics, general medicine and general surgery, District Hospitals (DHs) with 200-350 beds and ten clinical specialties and Medical Colleges and the General Government Hospitals (GGHs), being large facilities providing teaching along with the medical services. All facilities are classified as 1, 2, 3, 4 with 1 being the lowest. For a mandal with more than one facility, the facility with the highest order is considered.

2. Number of facilities (*N*_*F*_) within a mandal.

3. Distance of a facility to the nearest main road or national highway (*D*_*R*_).

4. Number of main roads or national highways (*N*_*R*_) passing through a mandal.

5. Number of neighbors (*N*_*N*_) for each mandal.

In this exploratory study, it is assumed that a linear relation holds for the expectation of each incidence *I*_*x*_, *x *= *REF, DW *and *P*:(8)

where the coefficients *α*_*i *_are to be estimated. Initially, to decide upon model composition, contribution of each variable was explored by using ordinary linear regression (OLS). Below, after model identification by identifying possible explanatory variables, an autoregressive approach is used to include spatial dependency in making a final estimate of the parameters. A spatial autoregressive modelling (SAR) is done for those variables that show a significant relation. A SAR model consists of a spatially lagged version of the incidence *I*_*x *_as(9)

where the matrix *W *represents neighbour relations, i.e. *w*_*ij *_= 1 if mandals i and j are neighbors, i.e. *dist*(*i, j*) < 50 km and *w*_*ij *_= 0 otherwise. The value of 50 km is used as a balance between a sparse neighbourhood pattern and a full inclusion of all the neighboring mandals. Other values have been tested as well but did not show strong differences. The parameter *ρ *is the autoregressive parameter establishing autocorrelation and the  denotes independent noise. Model (8) is equivalent to (7), except for the neighbourhood structure and the autoregressive parameter *ρ*. The spatial weights matrix *W *is standardized such that its rows sum to 1 [23]. The 164 mandals having a PPTCT center were selected for the analysis, the other mandals were discarded. The distance of 50 km for neighbourhood definition resulted into 395 neighboring mandals.

All layers have been created in an ArcGIS environment. OLS has been done in SPSS [24], with one variable at a time and *I*_*REF*_, *I*_*DW *_and *I*_*P *_as the response variable, whereas the SAR analysis has been done using the spdep library in the R package [25].

## Results

### Outlier analysis

**Figure 2:**
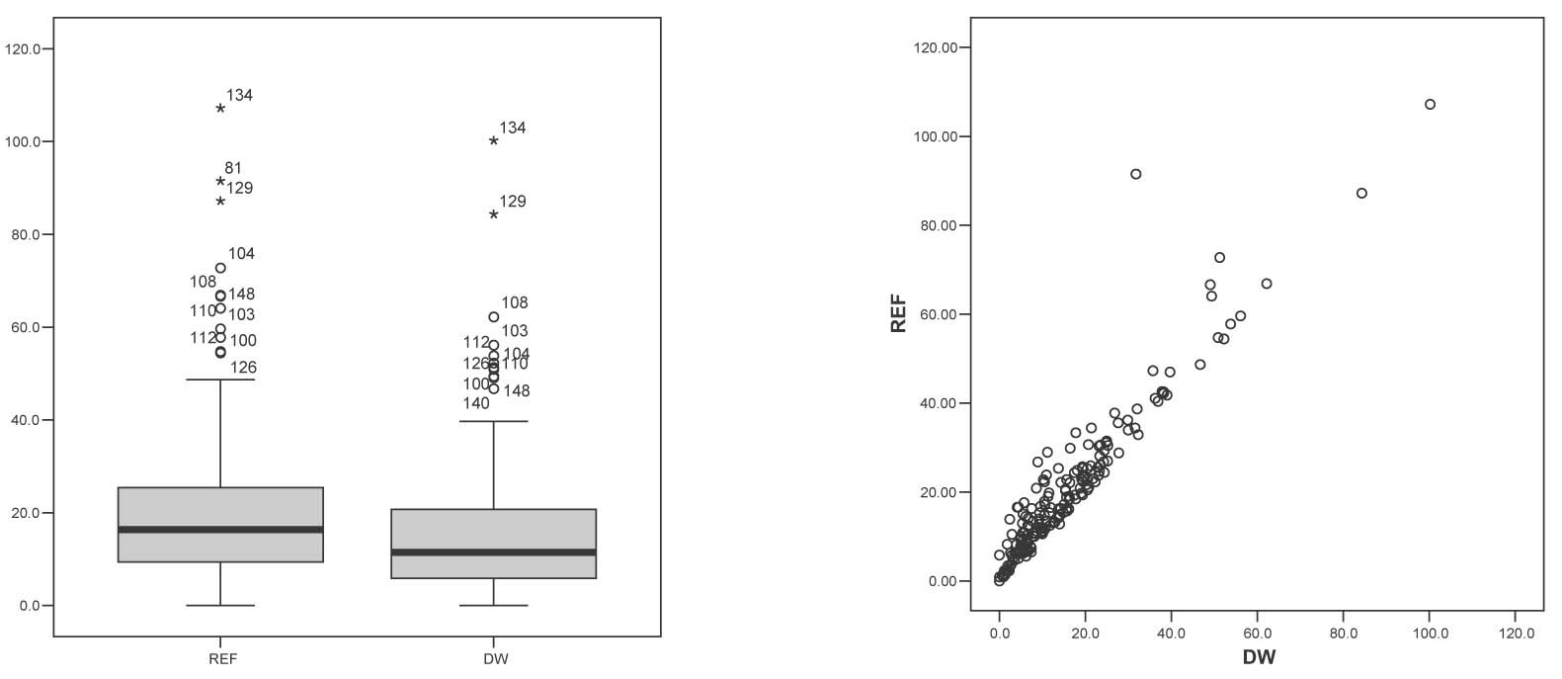
**incmandal**. Boxplots of *I*_*REF *_and *I*_*DW *_(*left*) and scatter plot of *I*_*REF *_*vs*. *I*_*DW *_(right), showing that *I*_*REF *_>*I*_*DW*_.

Figure 1 shows *I*_*REF*_, *I*_*DW *_and *I*_*P *_maps. Patterns of spread displayed by *I*_*REF *_and *I*_*DW *_are largely similar, both showing a higher incidence in the coastal edge of Andhra Pradesh and around the state capital Hyderabad than in the rural areas within the state. *I*_*P *_is lower than either *I*_*REF *_and *I*_*DW*_, generally taking values below 15 with only 4.8% of the mandals having an incidence between 15 and 22. Also, *I*_*P *_is distributed more evenly over the state, than either *I*_*REF *_or *I*_*DW*_. *I*_*REF *_on the average is higher than *I*_*DW *_in almost all locations (Figure 2).

**Figure 3:**
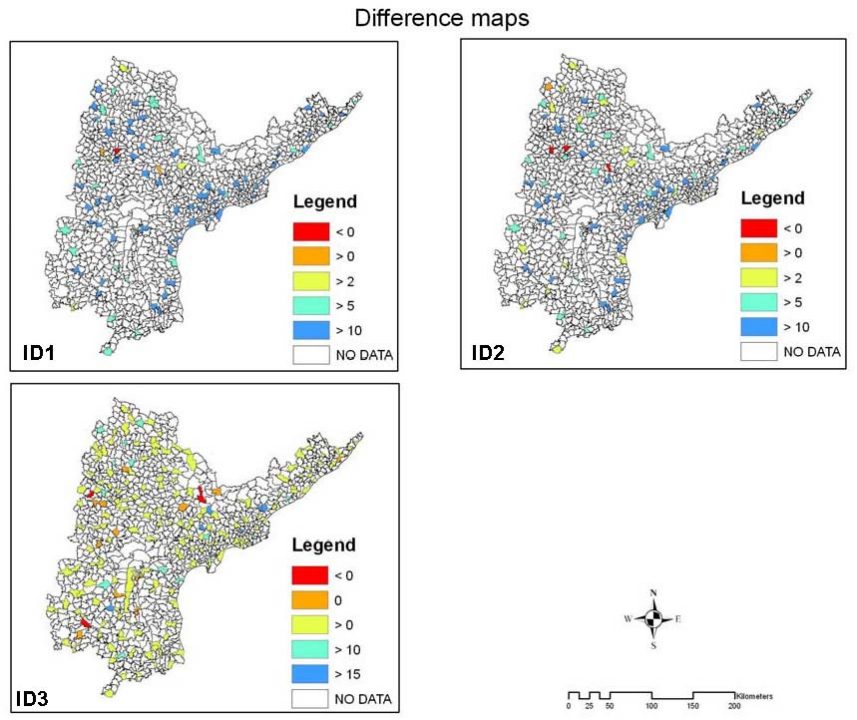
**diffs**. Difference maps calculated to understand the population mobility in age groups 15-39. In *ID*_1 _and *ID*_2 _red mandals are the locations where *I*_*P *_>*I*_*REF *_and *I*_*P *_>*I*_*DW*_, blue mandals are those having exceptionally higher *I*_*DW *_and *I*_*REF*_. In *ID*_3 _Rrd mandals have *I*_*DW *_>*I*_*REF*_, orange mandals have *I*_*DW *_= *I*_*REF *_green mandals have *I*_*DW *_<*I*_*REF *_and blue mandals have *I*_*DW *_<<*I*_*REF*_. Blue mandals show the places where the lowest number of *DW*s gets tested.

*ID*_1_, *ID*_2 _and *ID*_3 _maps represent the mandals that explain movement of HIV-positives (Figure 3). Assuming that incidence for pregnant women *I*_*P *_is generally lower than incidence for the general population, as they are a subsection of the whole female population, it is noted that HIV-positive females are apparently moving from mandals with negative values and values up to 2 to other mandals for getting tested. Such an approximating approach provides a clue in understanding the differences in the incidence in these mandals. In *ID*_3 _the interest is in the end values as these are the places which have either a higher *I*_*REF *_value or a higher *I*_*DW *_value. At mandals unaffected by movement, *I*_*REF *_and *I*_*DW *_should be equal. Hence a much higher value for either of the two represents a mandal with females either moving in or out for testing.

### Spatial Cluster analysis

**Figure 4:**
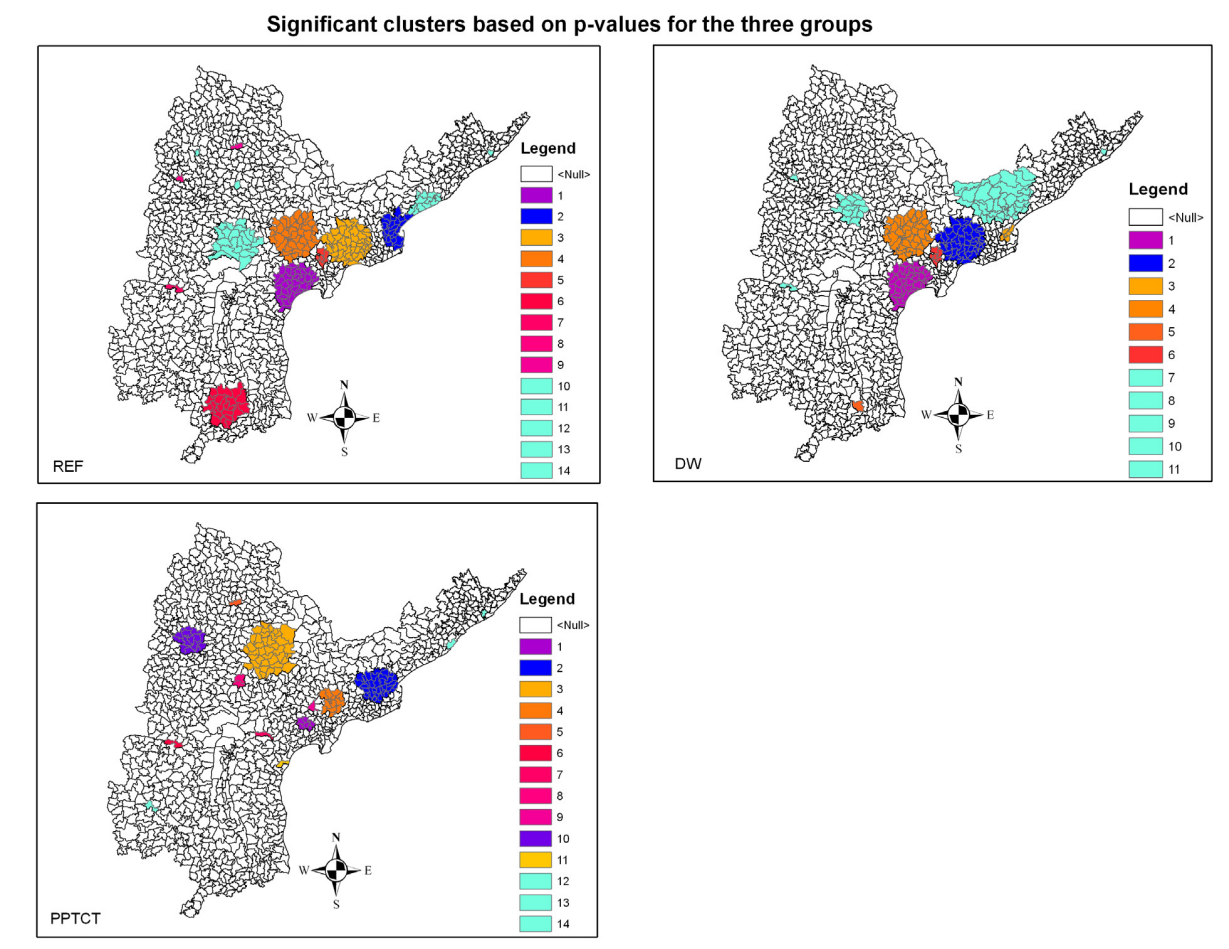
**clusters**. Results of cluster analysis showing the significant clusters for *I*_*REF*_, *I*_*DW *_and *I*_*P*_. Clusters shown in cyan are non-significant ones as based on the *p*-values.

Cluster analysis is performed to draw regions in the three classes which represent high rates of incidence. Figure 4 shows the results of the cluster maps for *I*_*REF*_, *I*_*DW *_and *I*_*P*_. Such clusters identify the mandals at higher risks as compared to their neighbors, including their statistical significance. The search radius for the moving window was kept at 5% of the population. Cluster analysis for *REF*s resulted into 14 clusters of which 9 were significant, for *DW*s into 11 clusters of which 6 were significant and for pregnant women into 14 clusters of which 11 were significant. The *DW*s are significantly clustered only at the SE coastal zone, a pattern which can also be witnessed in the incidence maps (Figure 1). As expected, both *REF*s and pregnant women are spread more equally over the state though in varying proportions.

### Establishing spatial relationships

**Figure 5:**
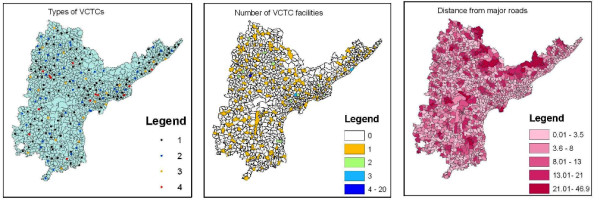
**layersA**. Layers of the explanatory variables generated for establishing spatial relations.

**Figure 6:**
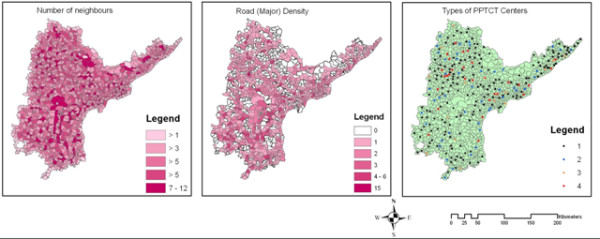
**layersB**. Layers of the explanatory variables generated for establishing spatial relations.

Relations of the spatial pattern of spread were modeled with underlying factors. Layers of the explanatory variables are shown in Figures 5 and 6. First the relationship between the spatial pattern was explored, outliers and the explanatory variables by means of visualisation. The hypothesis is that bigger facilities would attract more HIV-positives, but it is seen that usually the locations with higher incidences have a smaller facilities, such as a CHC. Similar overlays were prepared for the cluster maps with other layers like distance from roads, number of facilities, road density and number of neighbors. The overlay analysis of the incidence maps of the three categories with the cluster maps and the difference maps was done.

Relations between *REF*s and pregnant women with the type of facilities and the *DW*s with the roads were explored. it is observed that a relation between the type of facilities and the pregnant women as CHCs usually have higher incidences, although a significant relation between *REF*s or *DW*s with higher order facilities was not discovered. The number of neighbors (*N*_*N*_) seems to affect incidence on the basis of the visual comparison. The distance from roads (*D*_*R*_) shows a relation to incidences displayed by the difference maps, although, these patterns are far from uniform.

To have statistical evidence, regression analysis was performed and the results are shown in table 2. Relatively low R^2 ^values ranging from (0 to 0.05), (0 to 0.07) and (0.01 to 0.3) for *I*_*REF*_, *I*_*DW *_and *I*_*P *_as the response variables respectively are observed. The highest R^2 ^value equal to 0.307 was observed for the relationship of the type of facilities with *I*_*P*_. The corresponding equation equals(10)

**Table 2:** OLS

Results of the OLS regression for establishing spatial relations.							
**Indicator**	**Const**	***N*_*N*_**	***N*_*F*_**	***T*_*F*_**	***D*_*R*_**	***N*_*R*_**	***R*^2^**

*I*_*REF*_	11.39	1.52					0.018

	20.44		-0.03				0.000

	13.32			4.24			0.054

	22.53				-0.50		0.023

	18.18					1.15	0.011

*I*_*DW*_	7.21	1.47					0.021

	15.85		0.05				0.000

	8.77			4.25			0.071

	17.10				-0.50		0.029

	13.53					1.22	0.016

*I*_*P*_	-1.45	0.85					0.067

	3.16		0.24				0.011

	-1.01			2.31			0.307

	4.27				-0.17		0.054

	1.78					0.90	0.090

This means that the incidence increases with 2.306 if the type of facility increases with one unit. All other variables do not significantly contribute to the incidence of any of the three categories. Using the OLS results, the SAR analysis was performed with IP as the response variable and *T*_*F*_. The following linear relation was found:(11)

and an estimated *ρ *parameter equal to 0.0359 (significant at the *α *= 0.05 level), hence with slightly different coefficients. Use of the conditional autoregressive (CAR) model model did not lead to any substantial change.

Finally relationships were established between *ID*_1 _and *ID*_2 _as a variable measured at the mandal level to the explanatory variables mentioned in section methods, applying a SAR analysis for quantification. The following model was found to be the best describing the variation in *ID*_1_:(12)

where the autoregressive parameter was estimated as 0.073 (*p *< 0.001) and an AIC value of 646.5. In this equation the contributions of *N*_*R *_is almost significant (p = 0.0897) whereas that of *N*_*F *_is not significant (*p *> 0.1). It shows that incidence in *REF*s is larger than in *P*s, and, although somewhat weakly, that this difference could be explained by road density, with a higher difference with an increasing road density. It was somewhat surprising, as initially the hypothesis that the most important explanatory variable would have been *N*_*F *_was not confirmed. Its consequences are also relevant for HIV treatment and follow-up. The next model to be the best describing the variation in *ID*_2_:(13)

where the autoregressive parameter *ρ *was estimated as 0.069 (*p *< 0.001) and an AIC value equal to 645.5 was obtained. In this equation the contributions of *N*_*R *_is almost significant (*p *= 0.062) whereas the other contributions are not significant (*p *> 0.1). It shows a positive relation between road density *N*_*R *_and differences in *I*_*DW *_and *I*_*P*_, as such supporting the initial hypothesis: the difference increases with increasing road density. This increase is larger for *REF*s than for *DW*s, in other words: *REF*s are more inclined to move to another mandal for being tested than *DW*s.

None of the variables unambiguously explains the behaviour of the type of tested females. Therefore, although it seems that females might be moving one cannot exactly capture the movement and the attributed reasons do not fully explain any of the hypothesized phenomena. The regions where the incidence in pregnant women is higher than the general population can be identified as the zones of movement and similarly those with high *DW*s; however no significance or a consistent cause could be attributed to this.

## Discussion

This empirical study presents a first step to capture the overall pattern of HIV incidence at the state level to address the movement of people for testing on HIV. Its consequences can be relevant for HIV treatment and follow-up.

Trend analysis by means of maps and graphs revealed that incidence in the referrals group, *I*_*REF*_, shows on the average higher values than incidence in the directly walking-in group, *I*_*DW*_. A possible explanation is that in India there is little movement among women. If women do not belong to the high risk groups, then infection occurs through their partners in marriage and they get tested as a *REF *instead of as a *DW*. This spatial pattern analysis also shows that *I*_*P *_is lower than *I*_*REF *_and *I*_*DW*_. The most likely explanation is that the number of HIV-positives from PPTCT centers represents only a fraction of the total female population. Several mandals, however, have larger *I*_*P *_than *I*_*REF *_and *I*_*DW *_values. With an underlying assumption that *I*_*P *_should be the lowest, the mandals defying the trend give us a reason to further explore potential causes. A hypothesis that this occurs at random should be tested against the alternative that a definite and clear cause exists, such as the quality of the unit and reported success stories. The current data set did not allow us to do so, however.

The higher rates of *I*_*REF *_and *I*_*DW *_in the South Eastern coastal zones are clearly shown, both by the spatial pattern analysis and by the cluster analysis. This area is marked with a dense highway network and active port business. According to [26], this is also a favourite destination for the female sex workers, most likely explaining the registered incidence in these areas. A clear distinction exists between mandals where people live, and mandals where their HIV status is recorded. Elevated clusters are found for *DW*s in this region whereas the pattern of *REF*s is more scattered. The high variation of *I*_*P *_in terms of spatial spread is caused by the fact that pregnant women are a control group which is supposed to reliably represent the underlying population. Also, a high incidence rate is observed in pregnant women almost all over Andhra Pradesh. The fraction of pregnant women is low in *REF*s and absent in *DW*s. These values therefore show that a relatively large number of HIV-positive women in the general population is either not getting tested or moves to another place. In particular, the South Eastern coast zone is attractive, being a well connected urban set-up. Other reasons for comparatively lower *I*_*REF *_and *I*_*DW *_values in the rest of Andhra Pradesh might be caused by the low testing rates and lack of adequate and easily accessible facilities.

The attempts to relate *I*_*REF*_, *I*_*DW *_and *I*_*P *_with different parameters reveal a few interesting correlations. *I*_*P *_shows a positive significant relation with the type of facilities. This is in accordance with the social behaviour where women using government facilities usually prefer higher order facilities for anti-natal care. Also, based on visual analysis, it is noticed that community health centers have often been associated with higher incidences of *REF*s and *DW*s. This means that it is not the hierarchy of facility based on size that plays a role but it is the presence of a facility. Therefore women are likely to get tested if a facility is present, either small or large, and if they are aware of it. Since no significant relationship was observed with the road infrastructure and the proximity, one can infer that it is not governed by the good connectivity whether women move for getting tested. This may also explain the assumption that capturing movement depends on the type of movement and the transportation modes available in a mandal.

The following recommendations are derived from this study:

• HIV is a dynamic disease and a good data capturing is the backbone of all the policies. Further analysis in a spatio-temporal domain may be the key to better understand the interplay of various factors.

• The fact that one can only partially, i.e. non-significantly, explain the relations of differences leads us to assume that at the scale of the study and the available data, much of the movement is random and that a more detailed data set should be collected to exactly identify where people are moving and what factors are governing them in their behaviour.

• From a policy point of view, it may be important to increase self-motivation among women specially belonging to the HRGs (High Risk groups) potentially represented by the DWs to get tested because of the rapid progressing of HIV. More focused and better policies are needed to enlighten women so that they do not wait for a reference but visit a VCTC to get tested. In particular Andhra Pradesh needs special attention to let women abstain from behaviour responsible for the spread, and to take special measures not to allow the disease to spread to other states.

• A better insight into the quality of the data may help to improve describing factors determining HIV spread and to support spatial decision making, like positioning new health care facilities.

• Common policy assumption of coincidence of residence and test place is challenged by the present study, and should be reconsidered in future policies.

The study was constrained due to some important factors. Different sources of data sets were used; hence interoperability is a major problem. Census data, administrative boundaries, NACO and road data all have different sources and different procurement time which have to be adjusted for each other. This loses the originality of data to an extent and hence affects the results. For this exploratory study the amount of available data was large, but still more could have been measured. Possibly, the use of additional information could lead to a better analysis with a higher amount of explained variation. The available data set, representative at the level of mandals, however, was already quite unique and as far as we know has not been analyzed before.

The aim of this study was to analyze the whole state of Andhra Pradesh, but since facilities are present only in a limited number of mandals the analysis addresses some 20% of the mandals. This is compensated by the fact that an analysis at the district level integrates data from many hospitals. The main point addressed in this study about HIV policy-making, however, is that a change is needed in a basic assumption that place of testing and residence coincide. Consequences of such divergence need to be further explored in future research. Data quality could further improve if a better registration is done. Women should deliver their home address when visiting a VCTC for being tested. Also, motivating information about their preference of choice should be provided.

## Conclusions

Some concrete conclusions follow from this study. First, it was hypothesized that higher order facilities would attract more HIV-positives, but the study shows that mandals with higher incidences usually have a lower order facilities, such as a community health center. Therefore a hypothesis for further research could be that anonymity attracts females to a lower order facility for testing. Second, a pattern is observed between the type of facilities and the pregnant women as community health centers usually had higher incidences. However, significant relation between *REF*s or *DW*s with higher order facilities could not be discovered. Finally, there is a significant relation between the incidence in pregnant women and the order of the testing center.

Several trends emerge from the present study. The outlier analysis and the cluster analysis show that women move for getting tested. The present dataset did not allow us to say where they move to and what the precise effect is on HIV registration. The assumption that there is a random movement is not traceable at the given scale, also because of the amount of missing data. Alternatively, movement is perhaps an interplay of other interacting socio-economic factors which need to be further addressed.

Further research involving more spatio-temporal data would be helpful. This study relies on the 2006 data, since only those had the detailed PPTCT information. The number of testing centers is increasing with time, and data from 2007, 2008 and 2009, with less missing values, might be used. Comparing different years may provide us with more conclusive inter-relationships. A next step may be to analyze *I*_*REF *_and *I*_*DW *_differences for males, either relating these incidence on the basis of assumptions to those of female incidences, or by using a different benchmarking. It would be interesting to explore the relationships of the male incidence with different variables. This together with the female analysis will give us a larger picture and better understanding of reasons for people to move and in the end more reliable HIV data of a better quality.

## List of abbreviations used

AH: Area Hospital; ANC: Anti Natal Clinic; CHC: Community Health Center; CAR: conditional Autoregression; DH: District Hospital; DWs: Direct Walkins; GGH: General Government Hospital; GIS: Geographic Information System; HIV: Human Immunodeficiency Virus; HRGs: High Risk groups; HSS: HIV Sentinel Surveillance; NACO: National AIDS Control Organization; NFHS: National Family Health Survey; OLS: Ordinary Linear Regression; PPTCT: Prevention of Parent to Child Transmission; REFs: Referrals; SAR: Spatial Autoregression; VCTC: Voluntary Counseling and Testing Centers.

## Competing interests

The authors declare that they have no competing interests.

## Authors' contributions

RK carried out the research, EA supervised the GIS activities, AS supervised the spatial statistics, GM took care of the social and HIV related issues, PKG and RDG helped in data collection. All authors have read and approved the final manuscript.

## Appendix 1

India, Said to Play Down AIDS, Has Many Fewer With Virus Than Thought, Study Finds New York Times - Asia Pacific section, June 8, 2007. This article contains also this authoritative quote about the drop of estimations in Kenya: This is a replay of what happened in Kenya, said Daniel Halperin, an expert on AIDS infection rates at the Harvard School of Public Health. When Kenya was more carefully surveyed in 2004, he said, its prevalence rate was halved, to 6.7 HIV/AIDS Cases In India Might Be Lower Than Current Estimates, Survey Says Medical News Today, 13 Jun 2007 and AIDS cases drop, but mostly due to revised data - Previous estimates of 39 million were inflated, global health officials say MSNCB via Associated Press, Nov. 19, 2007
